# Determining level of care appropriateness in the patient journey from acute care to rehabilitation

**DOI:** 10.1186/1472-6963-11-291

**Published:** 2011-10-31

**Authors:** Christopher J Poulos, Christopher Magee, Guy Bashford, Kathy Eagar

**Affiliations:** 1 Centre for Health Service Development, Building 234, iC Enterprise 1, Innovation Campus, University of Wollongong, NSW 2522, Australia; 2 Department of Rehabilitation Medicine, Port Kembla Hospital, PO Box 21, Warrawong, NSW 2502, Australia

**Keywords:** acute care, subacute care, rehabilitation, utilization review, casemix, patient selection, InterQual

## Abstract

**Background:**

The selection of patients for rehabilitation, and the timing of transfer from acute care, are important clinical decisions that impact on care quality and patient flow. This paper reports utilization review data on inpatients in acute care with stroke, hip fracture or elective joint replacement, and other inpatients referred for rehabilitation. It examines reasons why acute level of care criteria are not met and explores differences in decision making between acute care and rehabilitation teams around patient appropriateness and readiness for transfer.

**Methods:**

Cohort study of patients in a large acute referral hospital in Australia followed with the InterQual utilization review tool, modified to also include reasons why utilization criteria are not met. Additional data on team decision making about appropriateness for rehabilitation, and readiness for transfer, were collected on a subset of patients.

**Results:**

There were 696 episodes of care (7189 bed days). Days meeting acute level of care criteria were 56% (stroke, hip fracture and joint replacement patients) and 33% (other patients, from the time of referral). Most inappropriate days in acute care were due to delays in processes/scheduling (45%) or being more appropriate for rehabilitation or lower level of care (30%).

On the subset of patients, the acute care team and the utilization review tool deemed patients ready for rehabilitation transfer earlier than the rehabilitation team (means of 1.4, 1.3 and 4.0 days from the date of referral, respectively). From when deemed medically stable for transfer by the acute care team, 28% of patients became unstable. From when deemed stable by the rehabilitation team or utilization review, 9% and 11%, respectively, became unstable.

**Conclusions:**

A high proportion of patient days did not meet acute level of care criteria, due predominantly to inefficiencies in care processes, or to patients being more appropriate for an alternative level of care, including rehabilitation. The rehabilitation team was the most accurate in determining ongoing medical stability, but at the cost of a longer acute stay.

To avoid inpatients remaining in acute care in a state of 'terra nullius', clinical models which provide rehabilitation within acute care, and more efficient movement to a rehabilitation setting, is required. Utilization review could have a decision support role in the determination of medical stability.

## Background

Changes to traditional models of care will be required if health systems are to manage the increasing demand that will be placed on hospitals as a result of an aging population [1-3]. One area where change may be necessary is the interface between acute care and rehabilitation. The selection of inpatients for rehabilitation, and the timing of transfer from acute care, are important clinical decisions that impact on quality of care and patient flow [4-6].

Inpatient rehabilitation is provided in almost equal quantities in the public and private sectors in Australia. Private sector rehabilitation is funded through a variety of private health insurance and accident compensation schemes. Public sector rehabilitation is funded by the states and territories with the funding including federal health grants. Most inpatients requiring rehabilitation receive a 'two-stage' model of care: acute care in an acute hospital followed by transfer for rehabilitation. Variables affecting the timing of transfer include the timing of the referral, the efficiency of the rehabilitation assessment process, patient stability and the degree of 'bed pressure' in both the acute and rehabilitation facilities. Whether the rehabilitation facility is co-located within the acute hospital, or 'stand-alone' in an off-site facility, also influences clinical decision making around patient selection and transfer [4,6].

The trend in Australia has been to locate inpatient rehabilitation services away from acute hospital campuses into small community hospitals when the latter can no longer provide safe, contemporary and efficient acute care [7]. While this has provided new roles for these hospitals, a downside is that patients may require a greater degree of medical stability prior to transfer due to the lack of acute and diagnostic support available. This may result in them remaining longer in acute care than might be the case if the rehabilitation facility were co-located with the acute hospital [5,6].

An exception to this two-stage model of care is the integrated stroke unit, a more contemporary clinical model that provides early rehabilitation for stroke patients, commencing in the acute hospital [8-11]. Patients requiring longer-term rehabilitation can then be transferred to a more suitable facility, while those able to be discharged directly from the stroke unit can receive ambulatory rehabilitation if required. However, not all acute hospitals have integrated stroke units and stroke represents less than 10% of inpatient rehabilitation episodes in Australia [12]. Further, it is not feasible to establish integrated acute/rehabilitation units for each of the myriad of impairments that patients receive rehabilitation for. Nor may it be necessary.

The two stage model is also reinforced by casemix (or activity-based) funding rules, which provide separate payments for the acute and the subacute episodes [13-17]. There is currently no casemix model in Australia that provides a payment for rehabilitation occurring in parallel with acute care, even though this may be the most appropriate clinical course. Examples where rehabilitation should ideally occur within acute care include times when the patient is able to participate in formal rehabilitation but is not medically stable enough to be transferred off-site, when the patient has to remain in acute care to undergo further investigations or procedures, or when there is a delay in accessing a rehabilitation bed.

During these periods the patient could be described as being in a state of *terra nullius *('land belonging to no one'), often designated by the acute care team as 'awaiting rehabilitation', with the team's attention diverted to higher acuity patients or to those who require therapy in connection with discharge directly home. Patients 'awaiting rehabilitation' often remain on the acute ward with minimal or no therapy [6,7]. Not only is this an unnecessary use of acute capacity, it may also contribute to further deconditioning and functional decline and prolong the subsequent rehabilitation episode.

Previous international and Australian research employing utilization review methodologies has shown that many acute hospital bed days do not meet the criteria for acute level of care, with many patients being deemed more appropriate for transfer to an alternate level of care instead [6,18,19]. Further, an Australian study which followed patients in acute care showed that utilization review criteria deemed patients 'appropriate' for rehabilitation or subacute care much earlier than did the rehabilitation service [6].

These findings raise questions about the nature of patients remaining in acute care when their need may be for rehabilitation, and about current models of care and payment models which allow this situation to arise. The optimal time for a patient to be transferred to rehabilitation and the implications of locating rehabilitation facilities away from acute hospital campuses need to be considered [4].

### Purpose of this paper

This paper reports utilization review data (using the InterQual^® ^utilization review tool) on a cohort of patients in a large regional acute referral hospital with a diagnosis of stroke, hip fracture or elective joint replacement, as well as other patients referred for rehabilitation transfer. It examines reasons why acute level of care criteria are not met for this cohort and, for a smaller cohort of patients, it also explores the differences in decision making between the acute care and rehabilitation teams around patient appropriateness and readiness for transfer. The utility of using a utilization review tool in a decision support capacity in this context is explored.

### The InterQual Criteria

The InterQual Level of Care Criteria is a proprietary product of the McKesson Corporation. They contain *admission*, *continuing stay *and *discharge review *criteria that match a patient's clinical status and services being received to levels of hospital care, including acute care and rehabilitation and subacute care, or to suitability for discharge home. The Adult (acute) Criteria contain clinical subsets grouped by body system or broad clinical categories, with each subset containing *severity of illness*, *intensity of service *and *discharge review *criteria. To meet appropriateness for *admission*, patients must meet severity of illness and intensity of service criteria. To meet appropriateness for *continuing stay*, only intensity of service criteria need to be met. When the patient is still in acute care, appropriateness for rehabilitation or subacute level of care is tested via *preadmission review *for these levels of care. To meet *preadmission *eligibility for a rehabilitation level of care, patients must satisfy criteria from five categories, with the content of the categories varying according to each clinical subset. The categories include criteria for: having had an illness, injury, surgery or exacerbation; having impairments requiring assistance; meeting clinical stability; having an ability to tolerate a rehabilitation program; and, not being able to be managed in a lower level of care than the one being tested. Further details of the content and application of the InterQual criteria can be found elsewhere [5,6,20,21].

## Methods

### Participants and procedure

All patients admitted in the acute hospital during the study period (30/4/2007 until 29/11/2007) with a diagnosis of stroke, hip fracture or joint replacement had InterQual utilization review criteria applied from admission (or surgery, in the case of joint replacement patients). These diagnoses were selected due to the higher likelihood that the patient would be referred for inpatient rehabilitation, thus allowing the capture of utilization review data from admission or surgery. All other patients in the acute care hospital referred for rehabilitation during the study period were also the subject of utilization review, but only from the date of rehabilitation referral.

The InterQual Adult (2006) (Acute and Rehabilitation/Subacute) Criteria were applied by clinical staff trained in its use and in the associated software (CareEnhance Review Manager - version 5.0). Patients with stroke, hip fracture or joint replacement were followed concurrently using the InterQual Adult (Acute) Criteria ('*admission' *then '*continuing stay' *criteria). All other patients referred for rehabilitation consultation were followed concurrently with the InterQual Adult (Acute) '*continuing stay' *criteria. The InterQual Criteria were applied on a daily basis until the patient no longer met criteria for continuing stay in acute care, at which point the '*discharge' *criteria were applied and the alternative level of care noted. If the patient met criteria for rehabilitation or other subacute level of care, '*preadmission' *criteria to confirm the level of care were applied. Patients continued to have '*continuing stay' *criteria applied on a daily basis until they were discharged home from the acute care hospital, transferred to rehabilitation, other hospital or aged care facility, or died. Reviewers applied the InterQual Criteria via daily review (or as otherwise specified within the Criteria) of the patient's medical record and observation and treatment charts, as well as by conferring with treating clinical teams when information was not readily available.

When criteria for acute level of care were not met, the reason was recorded. This was done via additional fields being created within the software. Throughout the study the rehabilitation service continued to use its in-house information management system, which recorded data relevant to the rehabilitation referral and consultation, including the dates of referral and consultation, consultation outcome, date ready for rehabilitation transfer and actual transfer date [22].

For a smaller cohort of patients (a convenience sample, based on reviewer availability, of patients referred for rehabilitation between the dates of 14/8/2007 and 17/11/2007), additional information was sought on the decisions of the acute care and rehabilitation teams about patient appropriateness for rehabilitation, and readiness for transfer. Data on the dates that the acute care and the rehabilitation teams deemed patients ready for rehabilitation transfer, the reasons why the rehabilitation team did not deem patients appropriate or ready for rehabilitation, and appropriateness for a rehabilitation alternative level of care according to utilization review, were collected by the utilization reviewers from information available in the medical record, the electronic data systems and from direct discussion with acute care and rehabilitation team clinicians.

### Data analysis

Data were extracted from the InterQual database and linked by patient medical record number with data from the hospital patient administration system and the rehabilitation service information system [22]. Linked data were analysed using Microsoft Excel (Microsoft Corporation, Redmond, Wash, USA), using descriptive statistics.

Ethical approval for the study was obtained from the Human Research and Ethics Committee of the University of Wollongong.

## Results

### Results on all patients to whom utilization review was applied

There were 696 acute care patient episodes representing a total of 7189 days in acute care. As shown in Table 1, the majority of patient episodes were the 'other rehabilitation' referrals (39.5%) followed by patients with stroke (20.8%), hip fracture (20.4%) and joint replacement (19.3%). Table 1 also provides information on gender and age.

**Table 1:** Characteristics of patients by episodes of care

	Stroke	Hip Fractures	Joint Replacement	Other Rehabilitation Referrals	Total
**Number of Episodes (%)**	145 (20.8)	142 (20.4)	134 (19.3)	275 (39.5)	696 (100.0)
**% Males**	43.4%	35.2%	38.1%	44.0%	40.9%
**% Females**	56.6%	64.8%	61.9%	56.0%	59.1%
**Average age years (SD)**	71.4 (14.8)	81.5 (9.2)	70.7 (10.2)	73.2 (13.1)	74.1 (12.8)
**Minimum Age (years)**	20.2	46.3	29.2	18.1	18.1
**Maximum Age (years)**	97.2	99.3	88.1	95.5	99.3

For the three patient types followed from acute admission or surgery, 56% of patient days in the acute hospital met InterQual criteria for acute level of care (see Table 2). The majority of hip fracture (55%) and joint replacement (71%) patient days of stay met criteria for acute level of care. For the 'other rehabilitation' group, 33% of days of stay met criteria for acute level of care from the time of rehabilitation referral.

**Table 2:** Patient days meeting InterQual Criteria for acute level of care, by diagnostic group.

Diagnostic group	No. of Patient Episodes	Days meeting criteria for acute level of care (no. [%])	Days not meeting criteria for acute level of care (no. [%])	Total days in acute care	Mean days in acute care^1 ^	Mean days of stay not meeting acute level of care^1 ^
Stroke	145	794 (49%)	843 (51%)	1637	11.3 (6.9)	5.8 (5.4)
Hip fracture	142	1011 (55%)	834 (45%)	1845	13.0 (8.5)	5.9 (6.6)
Joints	134	727 (71%)	299 (29%)	1026	7.7 (4.3)^2^	2.2 (2.8)
**Sub-total**	**421**	**2532 (56%)**	**1976 (44%)**	**4508**		
						
Other rehabilitation referrals	275	897 (33%)	1784 (67%)	2681	N/A^3^	6.5 (7.1)
**Total**	**696**	**3429**	**3760**	**7189**		

^1 ^Standard deviations provided in parentheses

^2 ^Mean days in acute care from the day of surgery

^3^No mean given as patients only followed from day of rehabilitation referral

When a day of stay did not meet InterQual Criteria for acute level of care, the main reason was identified. These data are shown in Table 3. Across all diagnostic groups, a delay in medical or other health professional consultation (17.9%) and an investigation or procedure delay (15.5%) were the most common reasons, followed by: patients being accepted, but not yet deemed ready by the rehabilitation team for rehabilitation transfer (12.9%); patients ready for transfer but awaiting a rehabilitation bed (12.6%); or patients awaiting transfer to an alternate level of care - ALOC - (12.3%).

**Table 3:** Reasons why days in acute care were deemed 'not appropriate', by diagnostic group

Reason	Stroke No. (%)	Hip # No. (%)	**Joint repl**. No. (%)	Other Rehab No. (%)	All Patient Days No. (%)
Delay: medical/allied health review	158 (18.7)	173 (20.7)	102 (34.1)	239 (13.4)	672 (17.9)
Delay: investigation/procedure	293 (34.8)	133 (16.0)	33 (11.0)	123 (6.9)	582 (15.5)
Accepted but not ready for rehabilitation	48 (5.7)	50 (6.0)	20 (6.7)	365 (20.5)	483 (12.9)
Awaiting ALOC: rehabilitation bed	56 (6.6)	117 (14.0)	28 (9.4)	272 (15.3)	473 (12.6)
Awaiting ALOC: other	88 (10.4)	151 (18.1)	4 (1.3)	218 (12.2)	461 (12.3)
Delay: rehabilitation consult or review	46 (5.5)	82 (9.8)	30 (10.0)	280 (15.7)	438 (11.7)
Unclear management plan	51 (6.1)	38 (4.6)	6 (2.0)	106 (5.9)	201 (5.4)
Delay in discharge home	25 (3.0)	21 (2.5)	35 (11.7)	119 (6.7)	200 (5.3)
No criteria/reasons outside of criteria	53 (6.3)	63 (7.6)	26 (8.7)	53 (3.0)	195 (5.2)
No reason identified	25 (3.0)	6 (0.7)	15 (5.0)	9 (0.5)	55 (1.5)
					
**Total**	**843 (100)**	**834 (100)**	**299 (100)**	**1784 (100)**	**3760 (100)**

There are some differences in the ordering of these reasons for each of the diagnostic groups. Delay in obtaining an investigation or procedure was the most common reason that stroke patient days did not meet criteria for acute care (34.8%), while for joint replacement patients the most common reason was a delay in obtaining medical or allied health review (34.1% of days). For the 'other rehabilitation' patients the most common reason was being accepted for rehabilitation, but not yet ready for transfer to an off-site facility (20.5%).

### Results on the subset of patients on whom additional information was collected

**Figure 1:**
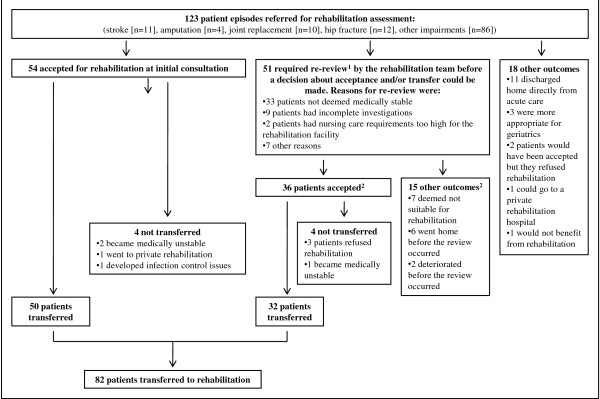
**Outcome of the 123 patient episodes referred for rehabilitation assessment**. ^1^80 patients were seen only once by the rehabilitation team, 35 had only one review, seven had 2 reviews and one patient required 3 reviews. Of patients requiring one or more further reviews, the mean time between initial consultation and the last review was 6.3 days (median of 5 days). ^2^Outcome of the re-review was that of the last re-review.

One hundred and twenty three patient episodes were included in this analysis. The mean age (76 years) and gender distribution (61% female) of these patients was similar to those rehabilitation referrals not included in the subset (mean age 76 years; 58% female). The diagnostic groups represented, and the outcomes following rehabilitation referral, are shown in Figure 1. Following the consultation/review process, 92 (75%) of the 123 patients who were referred for rehabilitation were, or would have been, accepted by the rehabilitation team. Eighty two patients (67%) were transferred. Reasons why patients were either not accepted for rehabilitation, or transferred, are shown in Figure 1.

Table 4 presents data on the 82 patients who were transferred to rehabilitation. It shows that, on average, the acute care team and the InterQual tool deemed the patient ready for rehabilitation transfer soon after referral (1.4 and 1.3 days, respectively), but that the rehabilitation team did not deem patients ready for transfer until some days later (mean of 4.0 days). The initial rehabilitation consultation occurred soon after referral (mean of 0.8 days), but there was a delay in effecting the rehabilitation transfer once the patient was deemed ready by the rehabilitation team.

**Table 4:** Subgroup analysis on patients transferred to rehabilitation (n = 82)

Period from rehabilitation referral until:	Mean (days)[SD]	Median (days)	Range (days)
The acute care team deemed patient ready for rehabilitation transfer	1.4 [3.1]	0	0 - 15
The InterQual tool deemed patient ready for rehabilitation transfer	1.3 [2.0]	0	0 - 9
The initial rehabilitation consultation occurred	0.8 [1.1]	0	0 - 7
The rehabilitation team deemed patient ready for rehabilitation transfer	4.0 [4.7]	2.0	0 - 28
The rehabilitation transfer actually occurred	5.7 [5.2]	4.0	0 - 28

### Medical stability

Using the InterQual Criteria as the standard measure of medical stability, the data were analysed to determine whether the patient subsequently became unstable in acute care after being deemed stable enough for rehabilitation transfer. Of the 82 patients transferred to rehabilitation 23 (28%) patients became unstable from the date the acute care team deemed them stable, compared with 7 (9%) who became unstable from the date the rehabilitation team deemed them stable. Nine (11%) patients became unstable from the time the InterQual tool deemed them ready for transfer.

Further, for patients deemed stable and ready for subacute care by InterQual on the day of initial rehabilitation consultation (n = 51), the rehabilitation team deemed those patients ready for transfer a mean 1.5 days (SD 2.6) after consultation (median 0 days, range: 0 to 12 days). However, for patients not deemed stable by the InterQual tool on the day of initial rehabilitation consultation (n = 31), then the rehabilitation team deemed those patients ready for transfer 6.0 days (SD 5.8) after consultation (median 5 days, range 0 to 27 days).

## Discussion

These data support previous findings from Australian and international studies showing that a large proportion of days of stay in acute hospitals do not meet utilization review criteria for acute level of care [5,6,19]. Patients with elective joint replacement had the lowest proportion of days not meeting acute criteria (29%), possibly reflecting the more predictable clinical pathway for this group. As might be expected, the 'other rehabilitation' group had the highest proportion of days not meeting acute criteria, having only been followed from the date of rehabilitation referral. They also had the highest average number of days per episode not meeting acute criteria.

The study adds to previous work reporting reasons for acute level of care criteria not being met. A Swiss study reported that delays in discharge processes accounted for 49% of inappropriate bed days, followed by delays in investigations, medical decision making and specialised consultations[23]. However, that study did not focus on patients who might be in need of rehabilitation. For the cohort of patients in the present study, the main reasons identified related to delays in processes and scheduling (waiting for clinical reviews, investigations or procedures) occurring within the acute hospital. Together, these accounted for about 45% of the inappropriate acute bed days and indicate that 'logistics' issues were a major impediment to patient flow.

Being more appropriate for transfer to rehabilitation or other lower level of care, or discharge home, were other key reasons why acute criteria were not met. Combined, these reasons accounted for about 30% of inappropriate bed days and indicate that patient flow from acute care may have been impeded by a lack of available alternate care settings and/or delays in facilitating transfer or discharge.

Not being ready for transfer to a rehabilitation facility, although accepted for rehabilitation, accounted for 12.9% of the inappropriate bed days. These days of stay did not meet utilization review criteria for acute level of care, and represent the discrepancy between the rehabilitation team and the utilization review tool in the determination of patient stability and readiness for transfer. This finding is consistent with prior research that found that patients wait a period of time in acute care when their need may be more appropriate for rehabilitation [6]. This issue was explored further in the second component of this study, and is discussed below.

The remainder of the inappropriate days of stay in acute care were due to a variety of reasons, broadly grouped as either an unclear management plan documented in the medical record or not having criteria available with which to approve the acute day. It is possible that some of these days might have been approved if the medical record was more comprehensive. Hospitals which depend on utilization review for funding decisions are reliant on the medical record providing sufficient clinical information for criteria to be met [24]. This is not the case in Australia, where formal utilization review is not conducted.

The second objective of this paper was to compare more closely, using a smaller cohort, the views of the referring acute care and rehabilitation teams on patient appropriateness for rehabilitation and the timing of transfer. The study found that there was not complete agreement between the teams on patient selection for rehabilitation. Clearer guidelines around the selection of patients will assist in patient flow and has the potential to improve patient outcomes [4,5].

Previous work using the InterQual tool has shown that the greatest time period in the 'referral-to-transfer-to-rehabilitation' process is that between the initial rehabilitation consultation and the day that the rehabilitation team deems the patient ready for rehabilitation transfer [6]. Consistent with these findings, the rehabilitation team in this study did not deem patients ready for transfer until some days after both the acute care team and the InterQual tool deemed patients stable for transfer (reported in Table 4). There was then a further delay in accessing the rehabilitation bed.

Determining medical stability for transfer to an off-site rehabilitation facility is an important aspect of patient care, for both patient safety and efficiency reasons. Transferring patients back to acute care from rehabilitation if they become unstable causes interruption to treatment programs, costs money in transportation and staffing, and adds to overall length of stay[20]. However, having patients wait for excessive periods in acute care until certain that they are stable can result in patients remaining in an acute bed when the more appropriate clinical need is rehabilitation [6,25].

An indicator of whether a patient was stable is to look at whether they become unstable after being deemed stable. In this study we used InterQual as an objective measure of medical stability to assess how the acute care team and the rehabilitation team compared in their determination of ongoing stability [18]. On this measure, the rehabilitation team performed better than the acute care team (9% versus 28% becoming unstable), however at the cost of much longer acute length of stay.

Despite the fact that the InterQual tool determined medical stability at about the same average time from referral as the acute team, but much earlier than the rehabilitation team, only 11% subsequently became unstable after InterQual determined readiness for transfer. This finding suggests that a utilization review tool such as InterQual could provide a more structured way for clinical staff to assess medical stability. This also seems logical, given that the tool provides a checklist of physiological and clinical indicators that must be met prior to recommending readiness for a lower level of care [5,6].

A decision support role for utilization review is also suggested by the finding that patients who were InterQual stable at initial rehabilitation consultation were deemed by the rehabilitation team to be ready for transfer earlier compared to those who were not InterQual stable at initial rehabilitation consultation (1.5 versus 6 days). The tool might therefore be helpful in identifying patients likely to be able to go to rehabilitation sooner, thereby assisting in planning patient flow. Future research could explore the role of utilization review in a decision support capacity to determine whether patients can be safely and appropriately identified and transferred for rehabilitation earlier in their acute course.

This study has a number of limitations. One of the limitations, in terms of generalizability, is that the rehabilitation facilities were all standalone, and therefore the requirement for medical stability prior to transfer will be greater than for facilities co-located within an acute hospital campus. However, this is a common scenario in Australia, and with health costs and pressures on acute hospitals rising, it is unlikely that there will be major changes to the location of rehabilitation facilities in the near future. Also, the study was conducted in a single large regional hospital, and so might not be generalizable to other institutions. The fact that the 'other rehabilitation' patients were only followed with concurrent utilization review from the time of rehabilitation referral limits the ability to compare this group with the groups followed from the time of admission or surgery. For this reason, the data from each of the diagnostic groups have been presented separately in the tables. However, the finding that the 'other rehabilitation' group still had the largest number of days of stay not meet utilization review appropriateness for acute care despite only being followed from referral suggests that this group needs to be examined further. Future research could follow a broader range of diagnostic groups with concurrent (or retrospective) utilization review from the time of admission, until rehabilitation transfer.

A further limitation of the study is that it has only served to identify and quantify the causes of inappropriate bed use. Future research could employ process analysis to further explore the underlying reasons why inappropriate bed use occurs and to test the effectiveness of process improvement techniques in reducing inappropriate bed use in acute care. Utilization review methods could then be used to verify the effectiveness of these interventions.

To overcome the problem of patients remaining in acute care in a state of *terra nullius*, other strategies need to be considered to ensure that they receive appropriate clinical care until ready and able to be transferred to rehabilitation [7,25]. Even if patients are not medically stable enough for off-site transfer, they may well be able to participate in rehabilitation. Early rehabilitation will help minimise the development of deconditioning and prevent the complications of bed rest, as well as allowing the planning necessary for complex patient discharge. Rehabilitation teams located in acute care are already in place in a pilot capacity in a few major acute hospitals in Sydney, Australia, funded under a new National government program [26]. To be sustainable, activity based funding models within Australia will need to be developed which allow for parallel rehabilitation care in the acute setting. However, if rehabilitation is commenced in acute care this could result in a longer wait for transfer to the actual rehabilitation unit for those patients, if they are not regarded as patients with the highest priority [27].

## Conclusions

In conclusion, this study supports the findings of previous research using concurrent utilization review to highlight potentially inappropriate acute care utilization. The study also found that, for this cohort, the main reasons for inappropriate acute care utilization were process inefficiencies within the acute hospital and delays in patients being deemed ready for, and then accessing, rehabilitation or other lower levels of care. It also found that there was a lack of agreement between the acute care and the rehabilitation teams in the determination of medical stability sufficient for transfer to an off-site rehabilitation facility, and that the use of a utilization review tool could potentially improve the accuracy and timeliness of determining medical stability, thereby being useful in a decision support capacity.

## Competing interests

The authors declare that they have no competing interests.

## Authors' contributions

CP made the major contribution to the study in the form of conception and design, acquisition of data and supervision of the research team, analysis and interpretation of data and drafting of the manuscript. CM made a substantial contribution to the analysis of data and providing critical comment on the manuscript. GM contributed to the design of the study, assisted in the conduct of the study and in the interpretation of data, and provided critical comment on the manuscript. KE made a substantial contribution to the conception and design of the study and provided critical comment on the manuscript. All authors read and approved the final manuscript.

## Authors' information

CP and GB are both senior rehabilitation physicians in hospital practice and clinical academics with the University of Wollongong, Australia. During the time of the study CM was a research assistant with the Centre for Health Service Development, University of Wollongong. KE is Professor and Director of the Centre for Health Service Development, University of Wollongong. All authors read and approved the final manuscript.

## Pre-publication history

The pre-publication history for this paper can be accessed here:

http://www.biomedcentral.com/1472-6963/11/291/prepub
